# The effect of a brief social intervention on the examination results of UK medical students: a cluster randomised controlled trial

**DOI:** 10.1186/1472-6920-9-35

**Published:** 2009-06-24

**Authors:** Katherine Woolf, I Chris McManus, Deborah Gill, Jane Dacre

**Affiliations:** 1 Academic Centre for Medical Education (ACME), UCL Division of Medical Education (DoME), UCL Whittington Campus, 4th Floor Holborn Union Building, Highgate Hill, London, N19 5LW, UK

## Abstract

**Background:**

Ethnic minority (EM) medical students and doctors underperform academically, but little evidence exists on how to ameliorate the problem. Psychologists Cohen *et al*. recently demonstrated that a written self-affirmation intervention substantially improved EM adolescents' school grades several months later. Cohen *et al*.'s methods were replicated in the different setting of UK undergraduate medical education.

**Methods:**

All 348 Year 3 white (W) and EM students at one UK medical school were randomly allocated to an intervention condition (writing about one's own values) or a control condition (writing about another's values), via their tutor group. Students and assessors were blind to the existence of the study. Group comparisons on post-intervention written and OSCE (clinical) assessment scores adjusted for baseline written assessment scores were made using two-way analysis of covariance. All assessment scores were transformed to *z-*scores (mean = 0 standard deviation = 1) for ease of comparison. Comparisons between types of words used in essays were calculated using t-tests. The study was covered by University Ethics Committee guidelines.

**Results:**

Groups were statistically identical at baseline on demographic and psychological factors, and analysis was by intention to treat [intervention group EM n = 95, W n = 79; control group EM n = 77; W n = 84]. As predicted, there was a significant ethnicity by intervention interaction [F(4,334) = 5.74; p = 0.017] on the written assessment. Unexpectedly, this was due to decreased scores in the W intervention group [mean difference = 0.283; (95% CI = 0.093 to 0.474] not improved EM intervention group scores [mean difference = -0.060 (95% CI = -0.268 to 0.148)]. On the OSCE, both W and EM intervention groups outperformed controls [mean difference = 0.261; (95%CI = -0.047 to -0.476; p = 0.013)]. The intervention group used more optimistic words (p < 0.001) and more "I" and "self" pronouns in their essays (p < 0.001), whereas the control group used more "other" pronouns (p < 0.001) and more negations (p < 0.001).

**Discussion:**

Cohen *et al.'s *finding that a brief self-affirmation task narrowed the ethnic academic achievement gap was replicated on the written assessment but against expectations, this was due to reduced performance in the W group. On the OSCE, the intervention improved performance in both W and EM groups. In the intervention condition, participants tended to write about themselves and used more optimistic words than in the control group, indicating the task was completed as requested. The study shows that minimal interventions can have substantial educational outcomes several months later, which has implications for the multitude of seemingly trivial changes in teaching that are made on an everyday basis, whose consequences are never formally assessed.

## Background

Students from ethnic minority (EM) groups have been found to underperform academically in medical school [1-9] and postgraduate examinations [10-14] in the UK, USA and Australia. In fact, ethnic differences in attainment are prevalent throughout compulsory education [15-17] and are found across disciplines in UK Higher Education [18].

Despite the prevalence of the ethnic gap in attainment in medicine, medical educationalists have struggled to explain it, and there is scant evidence to support the use of any practical measures to ameliorate it. Some researchers have suggested the effect may be partially due to subtle linguistic differences between candidates and examiners [4,14]; however that does not explain differences on machine-marked written assessments [1-3]. Only a small part of the ethnic disparity in medical students can be explained in terms of prior educational underachievement or differences in other background variables [19].

Social psychologists in America have proposed that people from ethnic minority groups underachieve academically due to a psychological phenomenon called stereotype threat [20,21]. According to stereotype threat theory, in test situations members of negatively-stereotyped groups (e.g. black students) can feel sufficient anxiety at the prospect of fulfilling a negative stereotype about their group that they subsequently underperform (see [21] and [22] for reviews). Although much of the research on stereotype threat has been done with African American students, the negatively stereotyped group does not have to be black for stereotype threat to occur. Stereotype threat has shown to negatively affect general academic performance in Latinos in the USA [23], mathematics scores in women [24] and sporting performance in white (W) men [25].

Evidence suggests that the negative effects of stereotype threat can be reduced by changing individuals' perceptions of themselves, their ability and their potential. [26,27]. In a recent US study [28] psychologists Geoffrey Cohen and his colleagues randomly allocated adolescent white and black students to self-affirmation intervention and control conditions. In the self-affirmation condition students wrote a short reflective piece about a value which was most important to them; in the control condition students wrote a short reflective piece about a value which was *not *important to them but which might be important to someone else. black students in the intervention condition did significantly better in post-intervention assessments. No change was observed in the white students. The pre-intervention ethnic gap in attainment was thus narrowed by almost 40%. The self-affirmation task was theorised to bolster students' self-esteem and self-worth, thus protecting black students against stereotype threat and improving their grades. White students' lack of improvement was explained by their hypothesised lack of stereotype threat.

The positive effects of self-affirmation have been shown in university students as well as the school children in Cohen *et al*.'s study [26,27]. It therefore seemed appropriate to attempt to replicate Cohen *et al*.'s study in the different context of EM underperformance at a UK medical school, where the majority of the EM group is of Asian (Indian, Pakistani or Bangladeshi) ethnicity – a group which has previously been found to underperform in medical school assessments [2,3] – [see Additional File 1 and Additional File 2].

We carried out a prospective cluster randomised controlled trial to assess the effects of including a brief self-affirmation intervention in the medical school curriculum, using high stakes machine-marked written assessments and OSCE (Objective Structured Clinical Examination) assessments as the outcome measures. Our research question was "can a brief self-affirmation task reduce ethnic differences in attainment in medical school examinations?".

### Objective and hypotheses

The objective of the study was to reduce the gap between W and EM students' post-intervention assessment results. The study tested two main hypotheses:

1. A brief, written self-affirmation intervention will improve the end-of-year written and OSCE examination performance of EM Year 3 medical students at UCL medical school relative to their mid-term written examination performance;

2. The same self-affirmation intervention will not affect the performance of W Year 3 medical students on the same outcome measures.

The study also tested the hypothesis that the types of words used in the intervention and control group essays would differ.

## Methods

### Participants

Eligible participants were, at the individual level, all students who started Year 3 at one London medical school in academic year 2006/7 (n = 348). At the cluster level, all 12 Year 3 tutors were eligible to take part. The exclusion criterion at the individual level was studying on a course other than the standard medical degree (MBBS) course. There were no exclusion criteria at the cluster level.

Individual student self-reported ethnicity data were obtained from medical student records, where ethnicity is broken down into the following categories: white, white British, white Irish, white Other, black Caribbean, black African, Asian Indian, Asian Pakistani, Asian Bangladeshi, Chinese, Asian Other, Mixed white and black Caribbean, Mixed white and black African, Mixed white and Asian, Mixed Other, Other, Unknown, Information Refused. We categorised these into white ('white', 'white British', 'white Irish' and 'white Other') and ethnic minority (all other categories except 'Unknown' and 'Information Refused').

### Randomisation

Independently of the study, Year 3 students were randomly allocated by Medical School Administration, using the RAND formula in Microsoft Excel, to 24 professional development course (PDS) tutor-groups run by 12 tutors (approximately 14 students per tutor group). As part of the study, we randomly allocated six of the tutors to the intervention condition and six to the control condition by having a member of staff who was uninvolved in the study and uninvolved in the delivery of the course to pull their names from a hat. Cluster randomisation was necessary to prevent students in the same tutor group being in different intervention groups, which would threaten blinding, and prevent the normal running of the group.

### Procedures and Interventions

Students at this London medical school study a compulsory professional development module called the Professional Development Spine (PDS). As a part of the Year 3 PDS course in the academic year 2006/7, all students undertook four tutor-marked reflective writing exercises which were formatively assessed. The third of the four reflective exercises was used for the present study.

In April and May 2007 all students received instructions via email from the PDS administrator on how to complete their reflective exercise. The task in the intervention condition was designed to encourage students to self-affirm their values by reflecting on them; whereas in the control condition students reflected on the values of another person which were *different *to their own. All students received a list of example values, which were: 'Being clever or getting good grades'; 'Being a good communicator'; 'Being a good team worker'; 'Creativity'; 'Independence'; 'Living in the moment'; 'Membership in a social group (such as your community, racial group, or medical school society)'; 'Relationships with friends or family'; 'Religious values'; 'Sense of humour'. These were based on the values in the Cohen *et al*. study with the 'team worker' and 'communicator' values being chosen from the professional values contained in the UK General Medical Council document *Good Medical Practice*. [29].

Intervention group instructions:

"Please spend a few minutes thinking about an incident that made you proud of yourself and your values. Then spend about 15 minutes writing a few paragraphs describing the incident, describing your value(s) and then reflecting on the reasons that incident made you proud of your value(s)".

Control group instructions:

"Please spend a few minutes thinking about an incident that helped you to recognise the value(s) of another person which were different from your own. Then spend about 15 minutes writing a few paragraphs describing the incident, that person's value(s) and then reflecting on the reasons you think that person had that/those value(s)."

Students were required to complete their reflective exercise and return it via email to the PDS administrator, who forwarded it to the researchers and the appropriate tutor. As part of the course, tutors marked the exercises as 'suitable for submission to portfolio' or 'not suitable for submission to portfolio' depending on the degree of reflection shown in the exercise. Reflection was assessed using Gibb's "cycle of structured debriefing" as a framework [30]. As in the usual reflective practice sessions, a few of these submissions were chosen by tutors to be discussed in tutorials two weeks later. The tutor's marks were not used as outcome measures in the experiment.

### Outcome measures

The primary outcome measure was performance in post-intervention summative written assessments in August 2007, adjusted for pre-intervention summative written assessments in March 2007. The secondary outcome measure was performance in post-intervention summative objective structured clinical examination (OSCE) assessment in August 2007, adjusted for pre-intervention summative written assessment in March 2007. The tertiary outcome measure was the number of types of words used in the reflective essays by the different groups. All pertained to the individual level.

### Written assessments

In 2006/7, Year 3 of the MBBS course at this London medical school had four clinical modules, with students sitting a mid-term summative written assessment in March 2007 after their first two clinical modules and an end-of-year summative written assessment in August 2007 after their remaining two clinical modules. Each written assessment consisted of two types of paper: one measuring generic clinical knowledge, the other measuring knowledge specific to the two modules most recently studied. The generic knowledge papers used an extended matching questions (EMQ) format, and the module papers used a single best answer (SBA) format.

At the beginning of the academic year, Medical School Administration divided students into two groups, which rotated around the modules in converse order. This meant that whilst all students regardless of group sat the first generic clinical knowledge paper in March and the second in August; students in different groups sat different versions of the module-specific papers at those times. To give an example, if Group 1 completed their orthopaedics rotation during the first two modules of the year they would sit a paper containing orthopaedics questions at the end of those modules in March. This means that Group 2 would therefore complete their orthopaedics rotation during their second two modules of the year and thus would sit a paper containing orthopaedics questions in August. These two March and August papers – whilst both measuring knowledge of orthopaedics – would, for educational reasons, contain slightly different questions which were designed to be of equivalent difficulty.

All written examinations were machine-marked using Speedwell software . Speedwell calculates reliability (internal consistency) using the Kuder Richardson Formula 20 [KR20 = *n*(σ_e_-Σ σ_r_)/σ_e _(*n*-1), where σ_e _is the variance of the candidate's score for the exam, Σ σ_r _is the sum of the variances of the candidate's scores for each response, and *n *is the number of responses]. The reliability of the written examinations ranged from 0.705 to 0.760 (see Table 1). This is sufficient to distinguish between groups, which was the purpose of this study.

**Table 1:** Reliability of the March 2007 and August 2007 generic and module-specific extended matching questions (EMQ) and single best answer (SBA) written examinations, calculated using the Kuder Richardson Formula (KR20)

**Examination subject**	**Date**	**Format**	**Reliability (KR20)**
Generic clinical knowledge	March	EMQ	0.758
	
	August	EMQ	0.730

Care of the Elderly and	March	SBA	0.760
	
General Medical Specialties	August	SBA	0.757

General Medicine, Medicine in the Community and Surgery	March	SBA	0.760
	
	August	SBA	0.705

### OSCE assessments

The OSCE was taken by all students at the end of the year over two days at the School's three clinical sites. It consisted of 15 five-minute stations which measured clinical and communication skills such as canulation, basic life support, systems examination and history taking. It used real patients, actor simulated patients and mannequins. At each station, candidates were marked by a single trained examiner who used a checklist to rate candidates' performance on individual station items as 'pass' 'borderline' or 'fail', and who also gave each candidate an overall global mark of 'clear pass' 'borderline pass' 'borderline fail' and 'clear fail'. The mark sheets were then machine-read using Speedwell which transformed these scorings into numerical marks. The standard was set using the borderline regression method [31]. The mean station/total score correlation for the examination was 0.897.

### Types of words used in the reflective essays

The frequencies of 53 types of word used in the reflective exercises submitted by each group were counted using Linguistic Inquiry and Word Count (LIWC) software [32]. LIWC groups words into four dimensions ('standard linguistic dimensions'; 'psychological processes'; 'relativity'; and 'personal concerns'). Each dimension contains between three and six categories (e.g. 'affective or emotional processes'; 'time') which themselves contain between four and seven subcategories (e.g. 'positive feelings'; 'past tense'). LIWC also provides a total word count, the number of words per sentence, and the percentage of words which are longer than 6 letters.

### Blinding: Students

Students were not informed of the existence of two separate conditions, and were blind to the existence of the study. They had already completed two reflective exercises as part of the course, so for this third exercise they were told in the email instructions:

"The instructions are slightly different for this block because we would like to know whether it is useful to ask students to reflect on particular subjects."

### Blinding: Assessors

The faculty members setting the Year 3 written assessments were blind to the existence of the study, and the written assessments were marked blind by machine.

### Blinding: Tutors

All but two of the twelve tutors (the reflective practice course leads) were blind to the study hypothesis and the outcome variable. Five months before the intervention all tutors were briefed that an experiment would be taking place, that they would be randomly allocated to one of two reflective exercise conditions, and that they should mark the exercises in the usual way. Tutors were told:

"All we ask is that you do not discuss the other condition with your group (e.g. if your group is asked to do the task in condition 1, please do not discuss the condition 2 task with them)."

Tutors were told that the rationale for the intervention was to investigate how students responded to being asked to reflect on particular topics.

### Statistical methods

All assessment results were transformed to *z-*scores [*z-*scores are Normally distributed with a mean of zero and a standard deviation of one. They are used here to take account of the fact that some students had taken different examinations to others as a result of being on different rotations]. The *z*-scores were then averaged and themselves converted to one pre-intervention baseline *z-*score, and one post-intervention *z-*score. A coefficient of intracluster correlation was analysed using Intercooled Stata 8.2 for Windows.

A two-way ethnicity by intervention analysis of covariance (ANCOVA) in SPSS v14 for Windows was used to compare W and EM intervention and control group scores on the primary outcome measure (post-intervention written assessment score corrected for pre-intervention written assessment score) and the secondary outcome measure (post-intervention OSCE score corrected for pre-intervention written score). Two-tailed p values < 0·05 were considered significant.

The frequency of types of words used in the essays of the intervention and control groups, and in the W and EM groups' essays (the tertiary outcome measure), were counted using LIWC software, and then compared using independent t-tests in SPSS v14 for Windows. Due to the number of tests performed, the level of statistical significance was set at p < 0.001.

### Ethical approval

The study met the requirements of the UCL Research Ethics committee, being exempt from formal ethical approval under the committee's exclusion conditions (see ) as it involved the analysis of routinely collected educational measures. Students were not informed of the study as the assignments were part of the normal educational process. However, with the agreement of the ethics committee, an e-mail had previously been sent to all students informing them that their assessment data may be used as the basis of research studies, and giving any who wished the opportunity to opt out of this process. None did so. The PDS lead and Reflective practice lead also agreed to the study. Reflective practice tutors were informed of the study's existence, and received a briefing report after the study was completed informing them of the aims, experimental hypotheses and results, and inviting them to feed back any comments to the research team.

### Details of funding

The study did not receive external funding.

## Results

There were no statistically significant differences between the intervention and control groups at baseline in terms of sex, ethnicity, age, possession of a previous higher degree, preclinical place of study, pre-intervention Year 3 written assessment scores, personality, study habits and stress (obtained by questionnaire as part another study conducted for KW's PhD). Individual participant and tutor characteristics are presented in Table 2 and described in the participants section above.

**Table 2:** Baseline information for each group at individual (student) and cluster (tutor) levels.

	**Intervention group n = (%)**	**Control group n = (%)**	**Total n = (%)**	**Group differences**	**p value**
**Total n = (%)Tutor factors at baseline**					

Total	6	6	**12**	n/a	n/a

male	1 (16.7)	2 (33.3)	**3 (25.0)**	n/a	n/a

white	6 (100.0)	5 (83.3)	**11 (91.7)**	n/a	n/a

**Student factors at baseline**					

Total	177/348 (50.9)	171/348 (49.1)	348 (100.0)		

Mean age	22 yrs 4 months	22 yrs 4 months	22 yrs 4 months	*t*(346) = 0.8	0.94

white*	80/175 (45.7)	87/166 (52.5)	167/341 (49.0)	χ^2 ^= 1.9; df = 3	0.60

Asian Indian, Pakistani, Bangladeshi	47/175 (26.9)	37/166 (22.3)	84/341 (34.0)	χ^2 ^= 1.9; df = 3	0.60

Chinese	16/175 (9.1)	12/166 (7.2)	28/341 (8.2)	χ^2 ^= 1.9; df = 3	0.60

All Other	32/175 (18.3)	30/166 (18.1)	62/341 (18.2)	χ^2 ^= 1.9; df = 3	0.60

Male**	69/176 (39.2)	59/171 (34.5)	128/347 (36.9)	χ^2 ^= 0.8; df = 1	0.36

Graduate entry***	23/176 (13.1)	20/166 (12.1)	43/342 (12.6)	χ^2 ^= 0.1; df = 1	0.75

With iBSC***	107/176 (60.8)	101/166 (60.8)	208/342 (60.8)	χ^2 ^= 0.2; df = 1	0.89

Oxford or Cambridge transfer	21/177 (11.9)	30/171 (17.5)	51/348 (14.7)	χ^2 ^= 2.2; df = 1	0.13

Mean pre-intervention written *z score*	0.05 (SD = 1.0)	-0.05 (SD = 1.0)	0.00 (SD = 1.0)	*t*(343) = -0.9	0.36

Mean Neuroticism score	8.1 (SD = 2.3)	7.8 (SD = 2.3)	8.0 (SD = 2.3)	*t*(270) = -0.8	0.45

Mean Conscientiousness score	11.3 (SD = 2.6)	11.3 (SD = 2.0)	11.3 (SD = 2.3)	*t*(272) = 0.2	0.86

Mean Openness score	11.1 (SD = 2.2)	10.9 (SD = 2.4)	11.0 (SD = 2.3)	*t*(272) = -0.9	0.39

Mean Agreeableness score	13.3 (SD = 1.6)	13.0 (SD = 1.6)	13.2 (SD = 1.6)	*t*(268) = -1.8	0.07

Mean Extraversion score	11.6 (SD = 2.1)	11.6 (SD = 1.8)	11.6 (SD = 1.9)	*t*(271) = -0.13	0.90

Mean Surface study score	14.9 (SD = 3.9)	14.7 (SD = 3.4)	14.8 (SD = 3.6)	*t*(265) = -0.5	0.61

Mean Strategic study score	18.5 (SD = 5.4)	17.7 (SD = 4.7)	18.1 (SD = 5.1)	*t*(266) = -0.8	0.45

Mean Deep study score	19.4 (SD = 4.1)	19.3 (SD = 3.9)	19.3 (SD = 4.0)	*t*(267) = -0.3	0.79

Mean GHQ (stress) score	11.4 (SD = 5.3)	10.2 (SD = 4.4)	10.8 (SD = 4.9)	*t*(262) = -1.9	0.06

* missing n = 7

** missing n = 1

*** missing n = 6 for graduate and iBSc combined (graduates do not take an iBSc)

**Figure 1:**
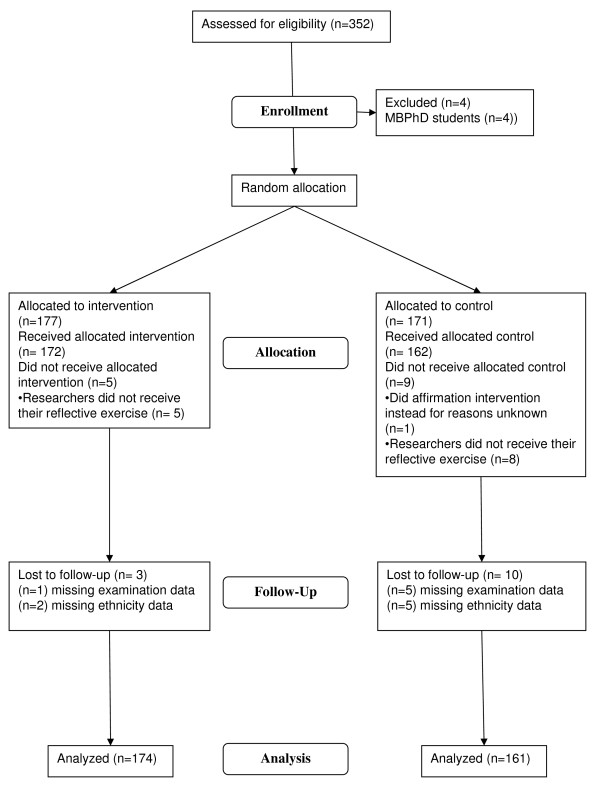
**CONSORT flow diagram showing the study profile**.

Figure 1 shows the trial profile. Data from 335/352 students were analysed (intervention condition n = 174; control condition n = 161): four students were not on the MBBS course, and 13 were lost to follow up (six with no August examination data and seven with no ethnicity data). All clusters were included in the analyses.

Data were analysed on an intention to treat basis, and we were aware of no important adverse events in the intervention group. The coefficient of intracluster correlation was found to be zero (95% CI: 0.00–0.03). The 95% confidence interval for the design effect was 1.00–1.82, which was smaller than 2 and therefore negligible. All subsequent analyses were therefore undertaken discounting the effects of the cluster or "nested" design. [33]. [see Additional file 1 for the effects of the intervention on the primary outcome measure presented by individual tutor group]. Mean scores with standard deviations for each group are given in Table 3.

**Table 3:** Means (standard deviations in parentheses) for each group on the primary and secondary outcome measures of post-intervention written z-score corrected for pre-intervention written z-score and post-intervention OSCE z-score corrected for pre-intervention written z-score.

**Ethnic group**	**Condition**	**Mean written** **z-score (SD)**	**Mean OSCE** **z-score (SD)**	**N**
W	Intervention	0.063 (0.90)	0.271 (0.96)	79
	
	Control	0.244 (1.00)	-0.002 (0.96)	84

EM	Intervention	-0.098 (1.09)	0.001 (1.00)	95
	
	Control	-0.175 (0.96)	-0.286 (0.97)	77

### Primary outcome measure: written assessment

**Figure 2:**
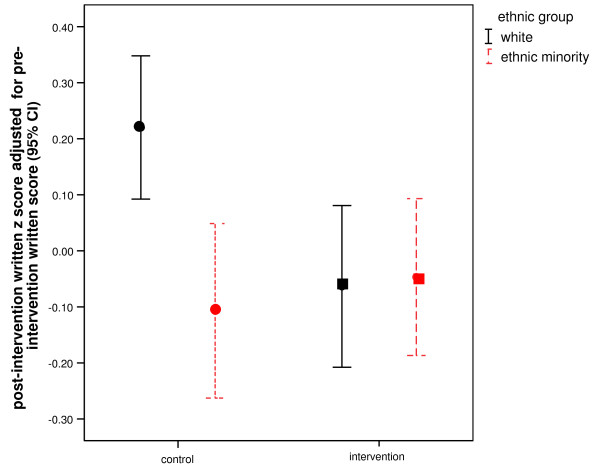
**The significant (p < 0.017) ethnicity by intervention interaction on adjusted post-intervention written assessment score, which was due to the significantly higher performance of the white control group (error bars with 95% confidence intervals)**.

The pre-intervention written and post-intervention written scores were highly and significantly correlated (r = 0.75, p < 0.001). Analysis of covariance of post-intervention performance with baseline performance as a continuous covariate (p < 0.001) showed a main effect of ethnicity, with W students [mean *z *= 0.078 (95% CI = -0.022 to 0.179)] achieving higher mean scores than EM students [mean *z *= -0.077 (95% CI = -1.176 to 0.022)] [F(4,334) = 4.64; p = 0.032]. There was no main effect of intervention (p = 0.121) but importantly, there was a significant ethnicity by intervention interaction [F(4,334) = 5.74; p = 0.017], which is shown in Figure 2 (Figure 2 shows the ethnicity by intervention interaction on the non-standardised residual of the post-intervention measure after taking baseline performance into account which is statistically equivalent to the analysis of covariance of post-intervention performance with baseline performance as a continuous covariate, i.e. post-intervention performance adjusted for pre-intervention performance).

*Post hoc *comparisons using the Ryan-Einot-Gabriel-Welsch procedure [34] confirmed that the four groups (W intervention, W control, EM intervention, EM control) performed significantly differently [F(3,334) = 5.76; p = 0.017], and the interaction effect was due to the W students in the control condition performing significantly better than all other groups [mean difference between control and intervention group scores in white group = 0.283 (95% CI = 0.093 to 0.474)], rather than improved EM intervention group performance [mean difference between control and intervention group scores in EM group = -0.060 (95% CI = -0.268 to 0.148)].

In terms of raw scores, W students in the control group achieved a mean mark that was approximately three points higher than that for EM students in the control group, whereas in the intervention condition, the ethnic difference in mean marks was only approximately 0.2 [see Additional file 1 for calculations of raw marks from *z-*scores, as well as an explanation for why the raw scores calculated from *z-*scores are approximations].

Two of the twelve tutors (one in the control group and one in the intervention group) were not blind to the nature of the study, but a formal comparison showed no evidence of a tutor knowledge × ethnicity × intervention interaction [F(1,334) = 0.049; p = 0.826] – [see Additional File 3].

### Secondary outcome measure: OSCE assessment

**Figure 3:**
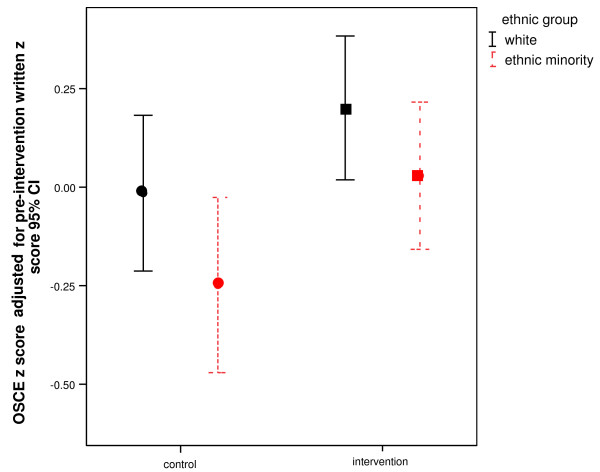
**The affirmation intervention significantly improved both white and ethnic minority performance on the OSCE z-score adjusted for baseline written z-score (p = 0.013)**.

The OSCE and pre-intervention written examination results were moderately correlated (r = 0.41; p < 0.001). Analysis of covariance of post-intervention OSCE performance with baseline written performance as a continuous covariate (p < 0.001) showed a main effect of intervention, with students in the intervention condition outperforming those in the control condition [mean difference = 0.261 (95%CI = -0.047 to -0.476); F(4,334) = 6.17; p = 0.013]. There was also a main effect of ethnicity, with W students achieving higher mean scores than EM students [mean difference *z *= 0.258 (95%CI = 0.472 to 0.044) F(4,334) = 4.18; p = 0.042]. The interaction term was non-significant [F(4,334) = 0.090; p = 0.76] and thus there was no indication that the intervention had particularly improved the EM students' performance. See Figure 3.

### Tertiary outcome measure: words used in the reflection exercise

#### Intervention and control groups

The intervention and control essays differed significantly in the types of words used (see Table 4). The intervention group used significantly more 'I' and 'Self' pronouns, whereas the control group used significantly more 'Other' pronouns. The intervention group also used more optimism words whereas the control groups also used significantly more negations and tentative words.

**Table 4:** Comparison between the numbers and types of words used in the control and intervention groups' essays.

**Dimensions**	**Word categories**	**Type of word**	**Group with highest frequency**	**Mean use by all students**	**Mean group difference (control – intervention)**	**P value**
Standard linguistic dimensions	Not applicable	*Word count*	*intervention*	*416.7*	-*19.2*	*0.34*
		
		*Words per sentence*	*control*	*26.9*	*0.27*	*0.74*
		
		% dictionary words	intervention	75.5	-1.1	0.04
		
		% words longer than 6 letters	control	21.2	1.7	0.001
		
		Total pronouns	intervention	10.9	-1.1	0.001
		
		**I (I, my, me)**	**intervention**	**5.0**	**-2.7**	**<0.001**
		
		**Self (I, we, me)**	**intervention**	**5.9**	**-2.6**	**<0.001**
		
		**Other (she, their, them)**	**control**	**3.3**	**1.4**	**<0.001**
		
		**Negations (no, never, not)**	**control**	**1.1**	**0.4**	**<0.001**
		
		Prepositions	intervention	14.4	-0.4	0.04
		
		Numbers	intervention	1.12	-0.3	0.001

Psychological processes	Affective/emotional processes	Total positive emotions	intervention	2.9	-0.3	0.02
		
		**Optimism and energy**	**intervention**	**0.9**	**-0.4**	**p < 0.001**
	
	Cognitive processes	Total cognitive processes	control	6.9	0.1	0.045
		
		**Causation (because, effect, hence)**	**control**	**1.1**	**0.4**	**<0.001**
			
		**Tentative (maybe, perhaps, guess)**	**control**	**1.8**	**0.5**	**<0.001**
	
	Sensory/perceptual processes	Hearing	intervention	0.9	-0.2	0.01
	
	Social processes	Total social processes	control	9.0	1.3	0.002
		
		**Other people references (1^st ^plural, 2^nd ^& 3^rd ^person pronouns)**	**control**	**2.5**	**0.2**	**<0.001**

Relativity	Time	**Total Time**	**intervention**	**6.2**	**-1.0**	**<0.0001**
		
		Past tense verb	intervention	6.6	-1.1	0.001
		
		Present tense verb	control	0.2	0.7	0.02
		
		**Future tense verb (will, might, shall)**	**control**	**1.0**	**0.3**	**<0.001**
	
	Space	**Down (down, below, under)**	**intervention**	**0.2**	**-0.1**	**<0.001**
		
		**Exclusive (but, except, without)**	**control**	**3.7**	**0.6**	**<0.001**
	
	Motion	**Motion (walk, move, go)**	**intervention**	**0.9**	**-0.4**	**<0.001**

Personal concerns	Occupation	Achieve	intervention	1.1	-0.3	0.005
	
	Leisure activity	Sports	intervention	0.4	-0.3	0.02
		
		Music	intervention	0.1	-0.1	0.05
	
	Metaphysical issues	Total metaphysical	control	0.2	0.2	0.015
		
		Religion	control	0.2	0.2	0.02

Examples of words given in parentheses. Only differences significant at the p < 0.05 level shown in the table (except word counts), and those significant at p < 0.001 in bold.

#### White and ethnic minority groups

As expected, W and EM students within conditions differed very little in the numbers of different types of words they used in their reflective exercises, only on 'hearing' words such as 'heard' 'listen' and 'sound' did EM students score significantly higher (see Table 5).

**Table 5:** Comparison between the numbers and types of words used in white (W) and ethnic minority (EM) students' essays.

**Dimensions**	**Word categories**	**Type of word**	**Group with highest frequency**	**t**	**Mean use by all students**	**Mean Difference between groups**	**p value**
Standard linguistic dimensions	% of dictionary words		EM	-2.0	75.5	-1.2	0.04
	
	Total pronouns	Pronoun	EM	-2.8	10.9	-0.9	0.01

Psychologi-cal processes	Affective or emotional processes	Optimism and energy (certainty, pride, win)	W	2.0	0.9	0.2	0.05
	
	Cognitive processes	Cognitive processes	EM	-2.2	0.1	-0.5	0.03
	
	**Sensory and perceptual processes**	**Hear (heard, listen, sound)**	**EM**	**-3.5**	**0.9**	**-0.3**	**<0.001**
	
	Social Processes	Communication (talk, share converse)	EM	-2.4	2.2	-0.4	0.02

Relativity	Motion	Motion (walk, move, go)	EM	-2.5	0.9	-0.2	0.01

Personal Concerns	Occupation	Job or work (employ, boss, career)	W	2.2	1.1	0.2	0.03
	
	Metaphysical issues	Religion (God, church, rabbi)	EM	-2.1	0.2	-0.2	0.04

Examples of words given in parentheses. Only differences significant at the p < 0.05 level shown in the table, and those significant at p < 0.001 in bold.

### Additional analyses

We provide a number of additional analyses [see Additional file 1]. These include: i) an analysis which shows that the ethnic difference in performance in this 2006/7 cohort of Year 3 students was similar in size to that in previous cohorts on the course [see Additional file 2]; ii) a graph which shows that effect of the intervention on W and EM students' performance on the primary outcome measure was not due individual tutor effects [see Additional file 3] iii) the results of a task which was designed to reinforce the experimental intervention and iv) a translation of *z-*scores back into marks. All analyses pertained to the individual level.

## Discussion and conclusion

This brief social intervention had significant effects on the written and clinical examination performance of Year 3 medical students three and a half months later, which highlights the necessity of research to systematically explore the potentially unexpected effects that clinical teaching may have on medical student performance.

The study was designed, as far as possible given the somewhat different context of medical school undergraduates, as a direct replication of the study by Cohen *et al*., with a clear *a priori *expectation of an ethnicity by intervention interaction in the same direction. This is indeed what we found on the main outcome measure of the written assessment. The implication being that ethnic differences in performance could in some way be mediated via social perceptions, and as a result might be altered by social interventions, and perhaps indeed by social interventions which are surprisingly minimal.

However, detailed *post hoc *comparisons of the means of the groups showed that the decrease in the ethnic gap was not due to increased performance of the ethnic minority students as hypothesised, but instead was due to a decreased performance of the white students in the intervention condition. The finding that the intervention reduced white students' performance was completely unexpected. The intervention was designed to build self-confidence and therefore should not have reduced performance in any group. These results also defy interpretation in terms of stereotype threat, particularly as white students generally tend to overperform in assessments [see Additional file 1]. In a further twist, the intervention improved the results of *both *ethnic groups on the secondary outcome measure of the OSCE.

The study benefited from a strong experimental design and theoretical underpinning – features that medical education research is sometimes accused of lacking [35]. The random allocation of individuals to clusters, and of clusters to conditions, increased confidence in the validity of the results, and ensured that the results were not due to differences on academic, demographic or psychological factors at baseline (as an additional check, baseline academic performance was adjusted for statistically). The results were probably not due to the clustered or "nested" design, as the design effect was calculated as negligible; and Figure 2 in the Additional material shows that the effect on the primary outcome measure was not due to tutor differences [see Additional file 3]. Neither were they likely to be due to demand characteristics [36] as the participants were blinded, and the word analysis provided further evidence that the students completed their exercises as instructed.

The unexpected results may relate to the characteristics of the study population. Most of the ethnic minority participants were Asian Indian, Pakistani or Bangladeshi ("South Asian") medical students, whereas those in the original Cohen *et al*. study were black African American teenagers. These two populations differ enormously on a great number of factors and it is therefore important to question how much, or indeed whether, stereotype threat applied to the ethnic minority students in this study.

Although pervasive negative stereotypes exist about the intelligence of people from black backgrounds [22,37,38], stereotypes about South Asians in educational contexts are perhaps less well known. Recent qualitative research has shown that a negative stereotype of Asian medical students may exist [39] which is similar to reported stereotypes of South Asian people as hard-working, rote learning, and apparently unwilling to mix with people who are not South Asian.[38,40,41] Moreover, although studies of UK higher education have shown that Asian Indian students tend to have a higher level of attainment at university than other ethnic minority groups, including blacks [17,18], they still has a lower record of achievement than whites throughout higher education, as well as specifically in undergraduate and postgraduate medical education.

This relative underachievement of Asian medical students, together with the existence of the negative stereotype together, mean that the ethnic minority group in this study might reasonably be expected to have suffered from stereotype threat. The degree of stereotype threat they might have been experiencing is however not known and cannot reliably be predicted. Future research could incorporate a measure of implicit stereotype activation both pre- and post-intervention to gain greater insight into the levels of stereotype threat in UK medical students.

The effect of the intervention on OSCE results may partially reflect the format of the examination. Unlike the written examinations, the OSCE is conducted face-to-face with the examiner, and scoring may be influenced by the way in which a candidate comes across both to the examiner and to the patients (simulated or real). Self-affirmations can increase positive feelings towards others such as love and connection [42] so students who reaffirmed their self-worth may have related better to examiners and patients and thus achieved higher scores.

The present study raises serious questions for medical educators (as well as social psychologists). The study was in many ways a success: the intervention was small and the effects were significant. And yet the outcomes were unexpected and difficult to explain. If the effects we had found were the results of a pharmacological or surgical intervention in patients, then a host of questions would have to be answered. We believe they also have to be answered here, not least by further replications with more and better controls, which would enable a meta-analytic review of the effects of this type of intervention on medical students' examination performance. If the examination behaviour of a robust group such as medical students is so sensitive to such tiny interventions then that is something that medical educators have to understand. In a commentary published with the Cohen *et al*. study, Wilson asked:

"Without the experimental results ... who would have thought that a 15-min exercise would have had such long-lasting effects"? [43]

That is indeed correct, and it also forces the deeper question of what other seemingly trivial fifteen-minute changes, casually made by teachers as a part of their daily activity, have effects that may actually be long-lasting and substantial in their consequences, but go unrecognised because they are not formally studied.

## Competing interests

The authors declare that they have no competing interests.

## Authors' contributions

KW and ICM conceived of the study. KW, ICM, DG and JD designed and implemented the study. KW and ICM analysed the data, and all authors interpreted the findings. ICM and KW wrote the first draft of the article and all authors revised it critically for important intellectual content. All authors approved of the final version.

## Pre-publication history

The pre-publication history for this paper can be accessed here:



## Supplementary Material

Additional file 1 **Additional analyses**. i) The first analysis shows that the ethnic difference in performance in this 2006/7 cohort of Year 3 students was similar in size to that in previous cohorts on the course [see Additional File 2]. ii) The second analysis shows that effect of the intervention on white and ethnic minority students' performance on the primary outcome measure was not due individual tutor effects [see Additional File 3]. iii) The third analysis shows the effects of a task which was designed to reinforce the experimental intervention. iv) An explanation of how z-scores relate to "real life" examination scores. Click here for file

Additional file 2 **Mean Year 1, 2 and 3 end-of-year assessment *z-*scores (± 1 standard error) for four cohorts of students who entered a London medical school in Years 2001, to 2004**. The figure shows that, in four cohorts of medical students, white students consistently outperformed ethnic minorities in Year 1, Year 2 and Year 3 examinations. Click here for file

Additional file 3 **White and ethnic minority students' mean performance, by tutor group (6 in the control condition, 6 in the intervention condition), on the primary outcome measure of post-written examination score adjusted for pre-intervention written examination score**. The figure shows that the effect of the intervention on examination scores was not due to tutor effects. Click here for file
