# Supplementary material for: The effect of a brief social intervention on the examination results of UK medical students: a cluster randomised controlled trial
Source: BMC Med Educ. 2009 Jun 24;9:35. doi: 10.1186/1472-6920-9-35 (PMC2717066; doi:10.1186/1472-6920-9-35)
Supplement: Additional file 1 — Additional analyses. i) The first analysis shows that the ethnic difference in performance in this 2006/7 cohort of Year 3 students was similar in size to that in previous cohorts on the course [see Additional File 2]. ii) The second analysis shows that effect of the intervention on white and ethnic minority students' performance on the primary outcome measure was not due individual tutor effects [see Additional File 3]. iii) The third analysis shows the effects of a task which was designed to reinforce the experimental intervention. iv) An explanation of how z-scores relate to "real life" examination scores. [file 1472-6920-9-35-S1.doc]

# Additional material

## i) Comparison of the 2006/7 cohort of Year 3 students with previous cohorts

Year 1, 2 and 3 end-of-year assessment results for four cohorts of students who entered one London medical school in 2001, 2002, 2003 or 2004 (N=320, 354, 319 and 314), were obtained from medical school records[[1]](#footnote-2). Because absolute marks differed between years, all marks within years were transformed into z-scores to allow direct comparison between years. Additional Figure 1 shows the mean marks (+ 1 standard error) of the students in Year 1, Year 2 and Year 3, classified by ethnicity. Analysis of variance found significant effects of ethnicity for Year 1, Year 2 and Year 3 marks (F(1,1286)=8.64, p=0.003; F(1,1245)=9.475, p=0.002; and F(1,1030)= 47.05, p<0.001). but no interactions of cohort with ethnicity (F(1,1286)=0.861, p=.461; F(1,1245)=0.234, p=0.873; and F(1,1030)=0.569, p=0.636), indicating that the ethnicity effect is stable across the four cohorts. Effect sizes expressed as Cohen’s *d* are -0.166 for Year 1, -0.177 for Year 2, and -0.438 for Year 3.[see Additional Figure 1].

## ii) Differences between tutors

In order to investigate whether the effect of the intervention on white and ethnic minority students’ performance on the primary outcome measure was due to differences between tutors, we calculated the mean score for white and ethnic minority students per tutor group. [see Additional Figure 2]. For clarity, standard errors of the mean are not shown, however all were approximately 0.25. The figure shows that the pattern of performance in all the control groups was virtually identical, with whites outperforming minority students. In the intervention group however, the pattern was for ethnic minority students' performance to be unchanged whereas white students’ performance was reduced.

## iii) Reinforcement of the intervention –rating the importance of the values

In order to increase the potential impact of the intervention, students were asked to indicate on a four point Likert-type scale their level of agreement with three statements concerning their selected value/s (“I strongly agree that this value is an important part of who I am” to “I strongly disagree that this value is an important part of who I am”). In the intervention condition these were, “In general, I try to live up to this value”; “This value is an important part of who I am”; “I care about this value.” The question in the control condition, although similar, focused on other people (“This/these value(s) influence some people”).

A mean of the four ratings given to the statements by students in the intervention condition was calculated and compared to the rating given to the statement by students in the control condition. Comparisons between White and ethnic minority students’ values were also made. Value data were missing for five students in the control condition and seven students in the intervention condition.

The mean in the intervention group was slightly higher than that in the control group, however overall students in the intervention condition agreed that the value they had reflected on related to themselves; and students in the control condition agreed that the value they had reflected on related to other people suggesting that the rating task reinforced the intervention approximately equally in each group. Similarly, the White and ethnic minority students similarly hardly differed in the ratings they gave the statements. The means and standard errors of the mean for the ethnic and intervention groups are shown in Additional Tables 1 and 2.

## iv) Translating z-scores back into raw marks

Whilst *z*-scores are mathematically useful, it is not always immediately obvious how they translate into the raw scores obtained by students. *z*-scores have a mean of 0 and a standard deviation of 1, therefore a *z*-score difference of 0.23 is equivalent to a score which is approximately a quarter of a standard deviation above the mean. It was not possible to translate this directly into a post-intervention written raw score due to the z-score being an amalgamation of various different scores (hence the purpose of the initial transformation); however to give a general idea of what a *z*-score of 0.23 means in terms of raw written exam scores, an example is given using the mean of all the mean raw written assessment scores, which are expressed as percentages (see Additional Table 2). The mean of the four written assessments (taken in March and August 2007) for all participants was 68.64, with a standard deviation of 8.34. Therefore a score which is 0.23 standard deviations above that mean is equivalent to a raw score of 70.56, which is greater than the overall mean of 68.64 by 0.23*8.34=1.92 raw marks, meaning a *z*-score of 0.23 is approximately equivalent to a score 2 raw percentage marks higher than the average. Thus, in the control condition White students in the scored approximately 3 percentage marks higher than ethnic minority students, whereas in the intervention condition they only scored approximately 0.2 percentage marks higher.

**Additional Figure 1: Mean Year 1, 2 and 3 end-of-year assessment z-scores (+ 1 standard error) for four cohorts of students who entered a London medical school in Years 2001, to 2004.**

**Additional Figure 2: White and ethnic minority students’ mean performance, by tutor group (6 in the control condition, 6 in the intervention condition), on the primary outcome measure of post-written examination score adjusted for pre-intervention written examination score.**

Additional Table 1: White and ethnic minority students’ agreement (mean, standard deviation, standard error of the mean, and 95% confidence intervals) with statements that the value they wrote about was important to them (intervention condition) or was important to other people (control condition)

| **Condition** | **Ethnic group** | **N** | **Mean** | **SD** | **SE** | **95% CI** |
| --- | --- | --- | --- | --- | --- | --- |
| Intervention | White | 71 | 3.62 | 0.39 | 0.047 | 3.53 to 3.71 |
|  | Ethnic minority | 89 | 3.59 | 0.46 | 0.049 | 3.50 to 3.69 |
| Control | White | 74 | 3.35 | 0.78 | 0.091 | 3.17 to 3.53 |
|  | Ethnic minority | 67 | 3.22 | 0.65 | 0.079 | 3.07 to 3.38 |

Additional Table 2: Means and standard deviations of raw written Year 3 assessment scores in 2006/7 (percent) . The appropriate average of standard deviations is calculated using the arithmetic mean on the variance scale

| **Written assessments** | **N** | **Range** | **Mean** | **SD** |
| --- | --- | --- | --- | --- |
| A1 | 345 | 40.0 to 90.7 | 70.98 | 9.09 |
| A2 | 342 | 48.0 to 86.7 | 72.81 | 7.76 |
| B1 | 342 | 31.0 to 82.8 | 64.28 | 8.45 |
| B2 | 345 | 40.0 to 85.0 | 66.47 | 8.00 |
| Mean | 344 | 39.8 to 86.3 | 68.64 | 8.34 |

1. For simplicity we have restricted this part of the analysis to those students who entered Year 1 of the UCL course. A proportion of students also enter the clinical course in Year 3, mostly from Cambridge, and they are omitted since they have no Year 1 or Year 2 results. It should also be noted that most UCL students intercalate a degree, but some do this after Year 2 and some during the clinical course. Not all of those in Year 2 therefore enter Year 3 in the immediately following year. [↑](#footnote-ref-2)
