# Supplementary figures and images for: The effect of a brief social intervention on the examination results of UK medical students: a cluster randomised controlled trial
Source: BMC Med Educ. 2009 Jun 24;9:35. doi: 10.1186/1472-6920-9-35 (PMC2717066; doi:10.1186/1472-6920-9-35)

## Slide 1
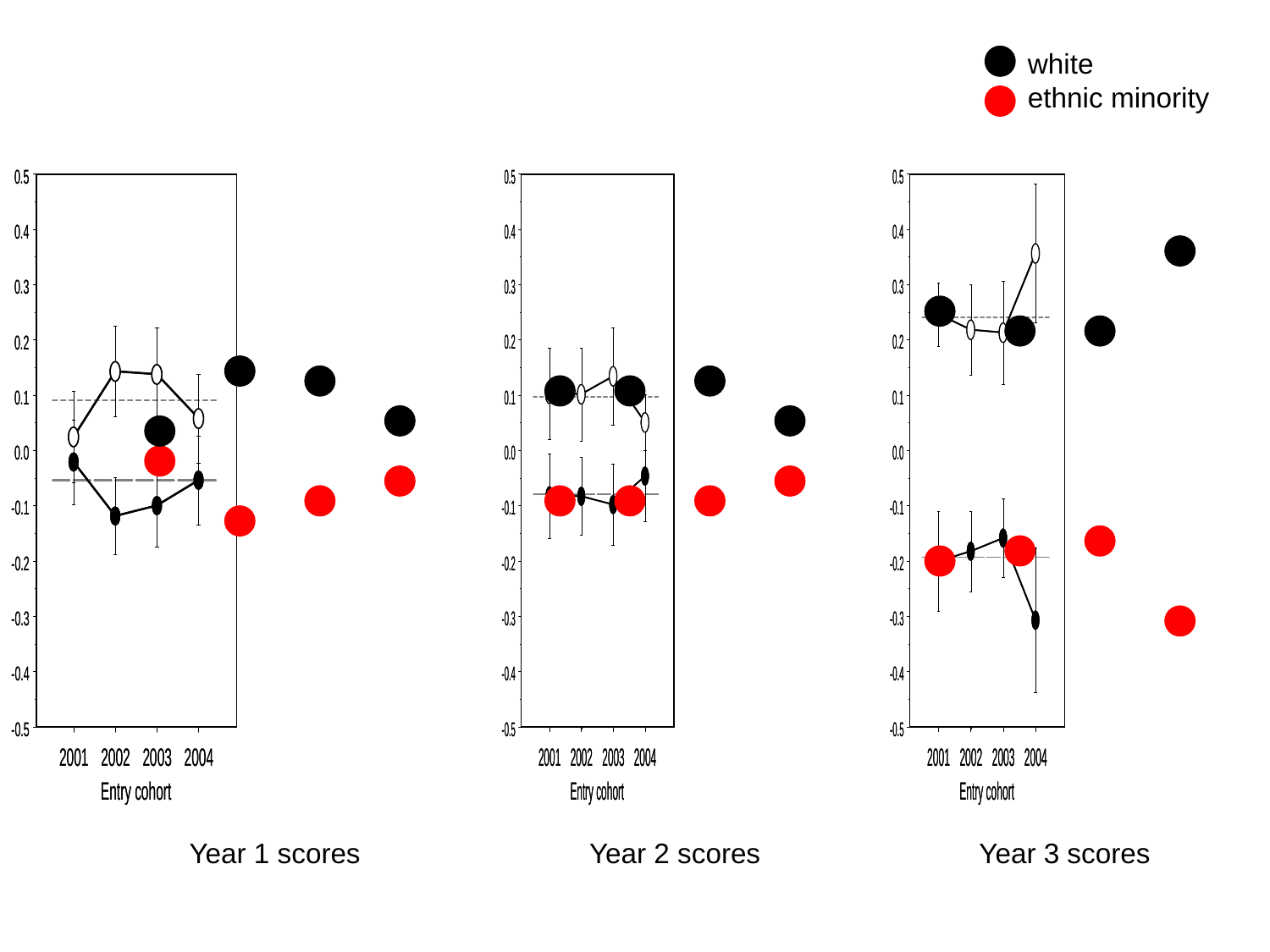

white
ethnic minority
Year 1 scores
Year 2 scores
Year 3 scores

Supplement: Additional file 2 — Mean Year 1, 2 and 3 end-of-year assessment z-scores (± 1 standard error) for four cohorts of students who entered a London medical school in Years 2001, to 2004. The figure shows that, in four cohorts of medical students, white students consistently outperformed ethnic minorities in Year 1, Year 2 and Year 3 examinations. [file 1472-6920-9-35-S2.ppt]
