# Supplementary material for: The effect of a brief social intervention on the examination results of UK medical students: a cluster randomised controlled trial
Source: BMC Med Educ. 2009 Jun 24;9:35. doi: 10.1186/1472-6920-9-35 (PMC2717066; doi:10.1186/1472-6920-9-35)
Supplement: Additional file 3 — White and ethnic minority students' mean performance, by tutor group (6 in the control condition, 6 in the intervention condition), on the primary outcome measure of post-written examination score adjusted for pre-intervention written examination score. The figure shows that the effect of the intervention on examination scores was not due to tutor effects. [file 1472-6920-9-35-S3.ppt]

## Slide 1
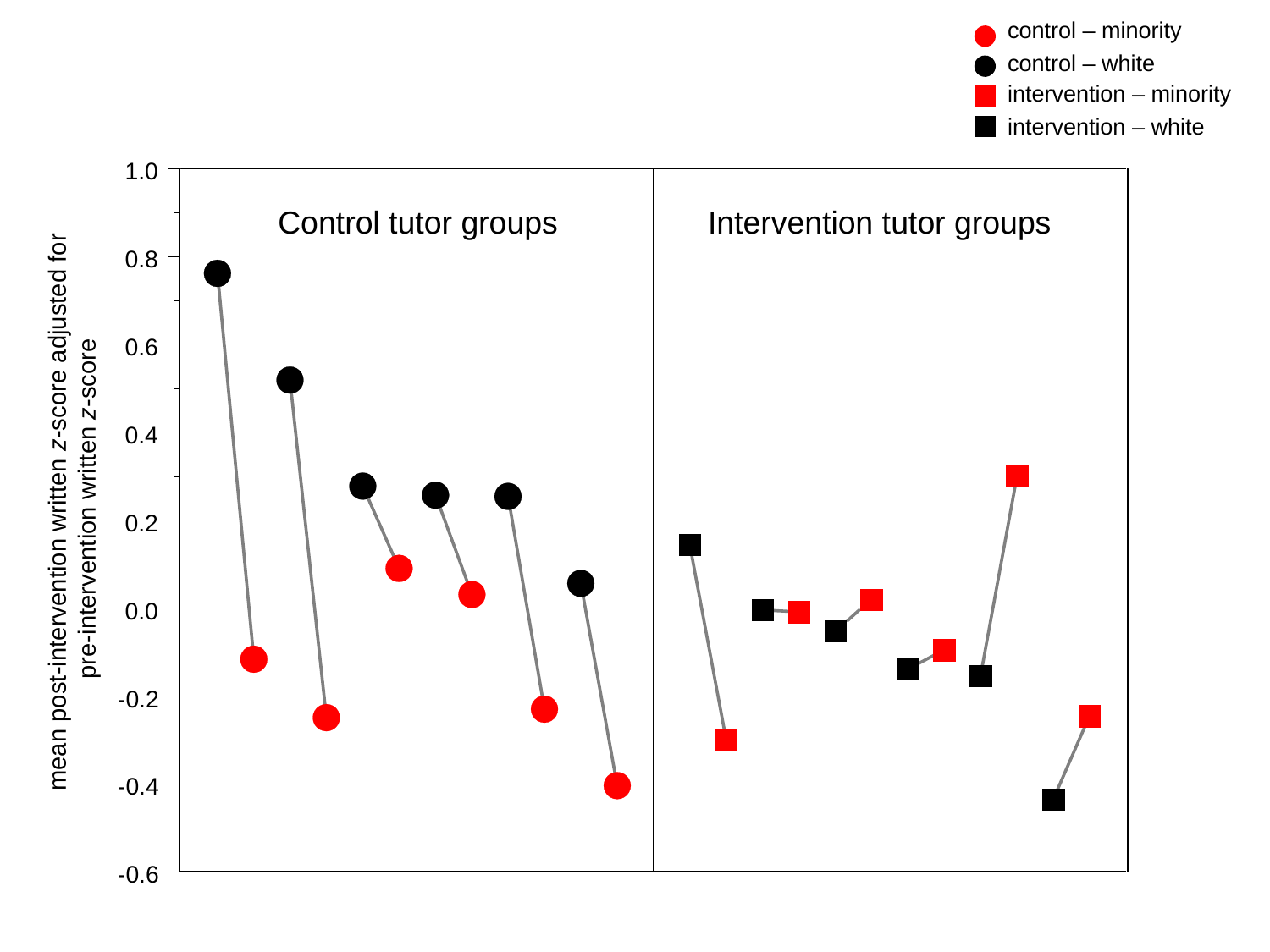

control – minority
control – white
intervention – minority
intervention – white
Control tutor groups
Intervention tutor groups
mean post-intervention written z-score adjusted for
pre-intervention written z-score
